# Oral Health in Individuals After Bariatric Surgery: A Systematic Scoping Review

**DOI:** 10.1007/s11695-025-07793-w

**Published:** 2025-03-19

**Authors:** Hisham Sindi, Sarah Almuzaini, Arwa Mubarak, Faisal F. Hakeem, Guglielmo Campus, Hani T. Fadel, Peter Lingström

**Affiliations:** 1 Department of Cariology, Institute of Odontology, Sahlgrenska Academy, University of Gothenburg, Gothenburg, Sweden; 2 Department of Preventive Dental Sciences, College of Dentistry, Taibah University, AlMadinah AlMunawwarah, Saudi Arabia; 3 College of Dentistry, Imam Abdulrahman Bin Faisal University, Dammam, Saudi Arabia; 4 College of Dentistry Hospital, Taibah University, AlMadinah AlMunawwarah, Saudi Arabia; 5 Centre for Epidemiology Versus Arthritis, The University of Manchester, Manchester, UK

**Keywords:** Bariatric surgery, Dental caries, Oral health, Periodontal disease, Tooth erosion

## Abstract

This scoping review appraises the available literature that investigates oral health after bariatric surgery (BS). Forty-two records were included (33 original studies and 9 systematic reviews). Bariatric surgery had a negative impact on the oral health related quality of life (three out of six studies). Relatively few studies found improvement following BS, probing pocket depth (2/10), clinical attachment level (1/9), bleeding on probing (3/10) and plaque (3/8). Conversely, BS was negatively associated with the DMFT (4/6), tooth wear (4/6) and oral radiographic findings (1/1). There was conflicting evidence regarding salivary parameters. To conclude, the literature on oral health in bariatric surgery patients is continuously growing. Positive as well as negative associations between oral health parameters and bariatric surgery are observed.

**Supplementary Information:**

The online version contains supplementary material available at 10.1007/s11695-025-07793-w.

## Introduction

Obesity is the abnormal excessive fat accumulation in the human body leading to health risks, usually with a BMI ≥ 30.0 [1]. It is directly associated with high mortality, morbidity and hospital expenses [2]. Bariatric surgery (BS) or weight loss surgery includes various procedures such as gastric band and gastric bypass surgery, and it is considered effective in managing obesity and promoting weight loss [1]. Gastric banding reduces the size of the stomach, while gastric bypass involves resecting part of the small intestine and re-routing to a small pouch of the stomach [1]. Recently, sleeve gastrectomy has gained popularity and involves removing a portion of the stomach [3].

According to the US National Institute of Health (NIH), BS is recommended for individuals with a body mass index (BMI) of 40 kg/m^2^ or those with a BMI of 35 kg/m^2^ who suffer from significant medical condition(s) [1]. Other factors, including fat distribution and actual adiposity, should also be evaluated before and after BS [4]. Following BS, considerable weight loss, recovery from type 2 diabetes mellitus, reduction of cardiovascular events and other health improvements are expected [1]. The improvement of psychological health may also occur after weight loss following BS [5]. Unfortunately, several adverse events have been reported, such as an increased risk for preterm birth [6], intestinal obstruction, anastomosis site complications and reflux symptoms [7]. In terms of oral health, a higher susceptibility to tooth decay, tooth erosion and increased salivary flow was also postulated and described in patients undergoing BS. Interestingly, results were divergent for the effect of BS on periodontal disease [8].

The relation of BS and oral health has focused mainly on the gastric bypass [3, 9–11]. The published systematic reviews on this topic highlights the need for a comprehensive overview of oral health in BS, particularly following the introduction of later BS techniques such the sleeve gastrectomy.

To fill this knowledge gap, we designed a scoping review to retrieve, synthesize and evaluate the oral health status of patients after BS. In contrast to systematic reviews, a scoping review tends to discuss the available body of literature related to a particular topic to when the literature is large and diverse [12].

## Methods

This scoping review was designed to broadly answer rising questions, fill out apparent knowledge gaps and highlight used methodologies pertaining to the topic of oral health and BS. This scoping review attempts to answer the following questions: (i) What are the study types that have been used to assess the oral health of individuals after BS? (ii) What oral health indicators have been studied in individuals who have treated with BS? (iii) What is the effect of BS on the individual’s oral health? and (iv) Is the risk to develop oral diseases increased in BS patients?

### PCC and Eligibility Criteria

#### Population

Individuals who have undergone different BS techniques.

#### Concept

Consideration of all oral health-related diseases and conditions from both self-reported (subjective) and objective examination and diagnostic tools.

#### Context

All experimental and observational studies including clinical trials, cohort, case–control studies and cross-sectional studies and systematic reviews were considered. Studies involved individuals who had undergone BS procedures such as gastric band, Roux-en-Y bypass and sleeve gastrectomy and who had the surgery once or more than once for cosmetic or medical purposes. Studies on taste perception, chewing behaviour or mastication kinematics, halitosis, case reports, case series and animal studies were excluded.

### Protocol and Registration

This review was performed in accordance with the Joanna Briggs Institute (JBI) methodology for scoping reviews [13]. It was drafted according to the Preferred Reporting Items for Systematic Reviews and Meta-analyses extension for scoping reviews (PRISMA-ScR) [14]. The study protocol was registered as a project in the Open Science Framework Registries on 04 January 2023 (https://osf.io/m8xb4/).

### Information Resources

An initial limited search in PubMed/MEDLINE, Scopus/Embase and Web of Science databases was undertaken to identify related articles and to assess the amount of available literature present. The text words contained in the titles and abstracts of relevant articles and the index terms describing them were used to develop a full search strategy in those databases. The search strategy, including all identified keywords and index terms, was adapted to be suitable for each selected database and/or information source. A snowball search for additional studies was applied to the reference lists of all included sources of evidence. The search strategy was based on the two main areas: BS and oral health. No restrictions were applied to publication year or language.

### Search Strategy and Evidence Selection

The search strategy was developed by author HS together with two librarians at the Biomedical Library, Sahlgrenska Academy, University of Gothenburg on 17 October 2022 (for full list of used search terms, see Appendix 1). The records were uploaded into the Rayyan web application for abstract screening (www.rayyan.ai). Authors SA and AM screened all titles and abstracts for eligibility independently after a pilot test by author HS. Selections were compared and conflicts resolved by author HS.

The search strategy was updated on 18 December 2023 by author HS and the two librarians at the Biomedical Library, Sahlgrenska Academy, University of Gothenburg using Bramer and Bain’s method for updating search strategies for systematic reviews [15] (Appendix 2). A third search for a second update of the search strategy was performed on 11 November 2024 using the same method mentioned previously [15] (Appendix 3). The procedure was repeated in uploading the new records into the web application. The same protocol was followed for title/abstract screening and full text reading. Reasons for exclusion of records in full text reading were recorded.

### Data Extraction Form

Data extraction was performed using an ad hoc form developed by authors HS and FFH, considering specific details including study population, context, method and key findings relevant to the scoping review questions. It was revised and modified as necessary during the process of data extraction. The authors were contacted to review, modify or discuss any disagreement where required. The final set contained information related to the publication including the title, year of publication, first author, country of study and study setting. The study design, objectives, inclusion criteria for the test and control groups (if any), age, sex and duration of the study were also included. Importantly, the BS technique, oral health variables and key findings related to the scoping review questions were established as a tool backbone.

### Data Analysis and Presentation

A narrative method was applied for data synthesis. Frequency of studies of different types, the association of oral health variables with BS and all other extracted data are provided (Appendices 4 and 5).

## Results

### Evidence Selection

**Fig. 1:**
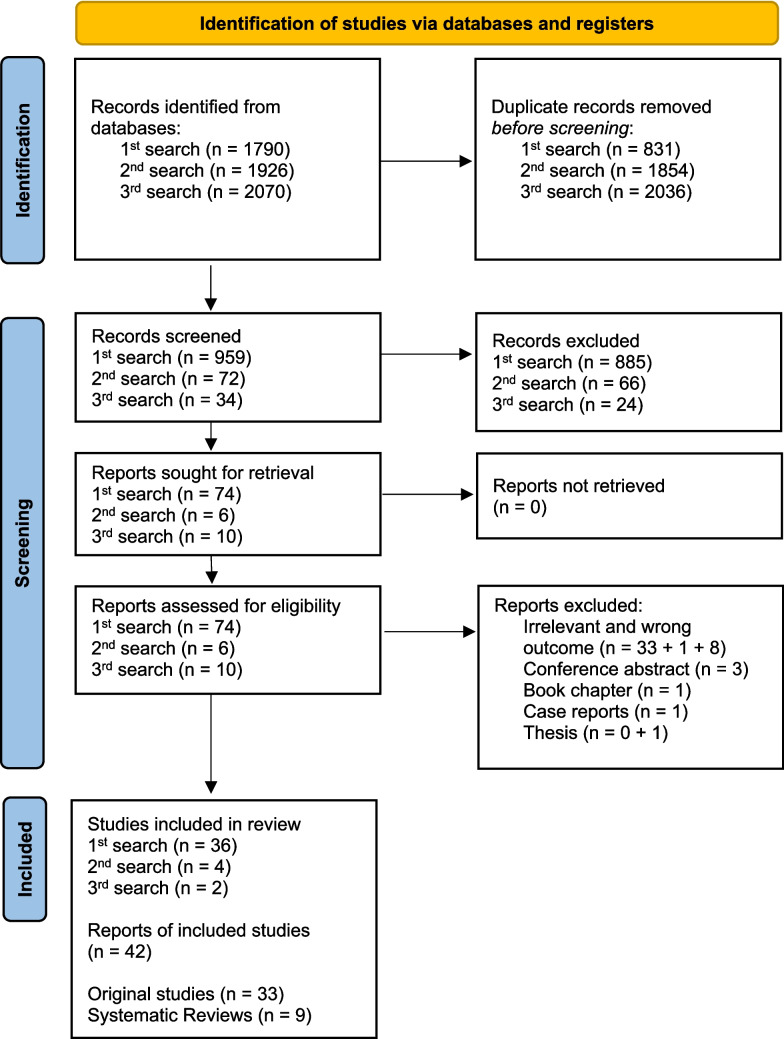
PRISMA flow diagram of the study selection process. *From:* Page MJ, McKenzie JE, Bossuyt PM, Boutron I, Hoffmann TC, Mulrow CD, et al. The PRISMA 2020 statement: an updated guideline for reporting systematic reviews. BMJ 2021;372:n71. https://doi.org/10.1136/bmj.n71 [73]

The initial first search conducted on 17 October 2022 produced 1790 records. After de-duplication, 959 records remained. The title/abstract screening resulted in 74 articles, which were included for eligibility assessment. Full text reading led to inclusion of only 36 articles. After deduplication of the second search held on 18 December 2023, 72 new articles appeared. The title/abstract screening in the updated search procedure resulted in six titles/abstracts included for eligibility assessment, out of which four articles remained following full text reading. The third search performed on 11 November 2024 resulted in 2 records after full text reading of 10 records following title/abstracts screening of 34 articles. Those 4 and 2 records, respectively, were added as an update to the previous 36 (Fig. 1). This led to a total of 42 records: 33 original studies and 9 systematic reviews, of which 6 were with meta-analyses.

### Publication Manner

**Fig. 2:**
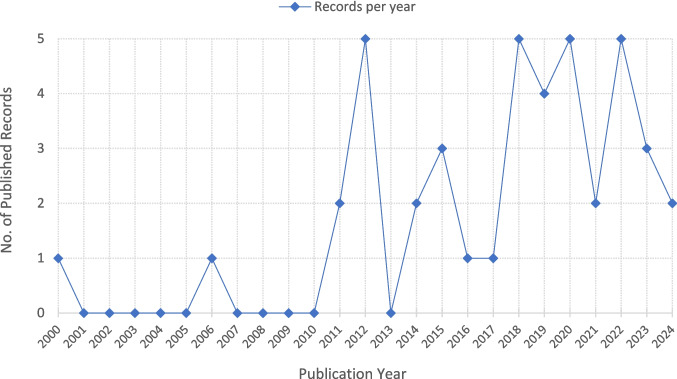
Line chart showing the number of retrieved published records each year during the specified data extraction period (*N* = 42)

The studies included within the specified time frame were published between 2011 and 2024, with the exception of two articles [16, 17] (Fig. 2). At least two studies were published per year in the above-mentioned range except in 2017 and 2021. Of the 33 original studies, 17 were conducted in Brazil, 4 in Sweden and 3 in USA (Table 1).

**Table 1:** Characteristics of the finally included original studies in the scoping review (*n* = 33)

Authors	Year	Study type	Study duration	Country of study	Study setting	Type of bariatric surgery	Number of participants	Age (years)	Sex (F%)	Studied variable category		Studied outcome(s)
										Subjective	Objective	
Greenway and Greenway [16]	2000	Phase I Cross-sectional Phase II Cross-sectional	-	USA	(Not mentioned)	JI Bypass^a^	Phase I Total = 23 Surgery (Ob/OW^b^) (average 10 years post op.^b^) = 18 Control (NW^b^) = 5 Phase II Total = 8 Surgery (Ob/OW) (25–30 years post op.) = 4 Control (NW) = 4	Phase I Surgery: < 30 (mean 25) Control: 24–40 Phase II Surgery: 36–56 (mean 48 ± 9) Control: 25–54 (Mean 42 ± 13)	(Not mentioned)	X	X	Phase I Surgery group only, before and after surgery: self-reported root surface caries Surgery and control groups: salivary chloride, bicarbonate and pH Phase II Surgery and control groups: stimulated salivary flow/secretion rate
Heling et al. [17]	2006	Cross-sectional	-	Israel	Medical clinic(s)/centre(s)/hospital(s)	SRVG^a^ Lap-Band^a^.SRVG	Total = 113 Surgery (1–10 years post op.) (Weight status not disclosed)	Mean 40 ± 10	75%	X		Self-reported oral hygiene practices, frequency of visits to the dentist, hypersensitivity of the teeth, dental treatment and sense of taste
Marsicano et al. [34]	2011	Prospective longitudinal single-group observational	6 months	Brazil	University hospital/academic departments	RYGB^a^	Total = 54 Surgery (Ob/OW) (before and 3 and 6 months post op.)	Mean 41 ± 10	81%	X	X	OIDP^c^ DMFT^c^, CPI^c^ and DWI^c^ Salivary flow/secretion rate
Valentine et al. [42]	2011	Prospective longitudinal single-group observational	12 months	USA	Medical clinic(s)/centre(s)/hospital(s)	LSG^a^ Lap-Band RYGB DS^a^	Total = 24 Surgery (Ob/OW) (before and 6 and 12 months post op.)	Mean 49 ± 8	100%	X	X	HRQoL^c^ questionnaire using the Medical Outcomes Study Health Survey (Short-Form 12, version 2) and salivary cortisol
Alves et al. [38]	2012	Cross-sectional	-	Brazil	University hospital/academic departments	(Not mentioned)	Total = 125 Surgery (Ob/OW) (≥ 6 months pot op.) = 41 Non-surgery (Ob/OW) = 42 Control (NW) = 42	Surgery Mean 43 ± 9 Non-surgery Mean 37 ± 10 Control Mean 33 ± 10	86%		X	BEWE^c^
Lakkis et al. [27]	2012	Controlled clinical	4–6 weeks	USA	University hospital/academic departments	(Not mentioned)	Total = 30 Surgery (Ob/OW) (≥ 6 months pot op.) = 15 Non-surgery (Ob/OW) = 15 (Intervention: non-surgical periodontal therapy)	Mean 47 ± 11	63%		X	PlI^c^, GI^c^, PPD^c^, BOP^c^ and CAL^c^
Netto et al. [40]	2012	Phase I Cross-sectional Phase II Prospective longitudinal single-group observational	2 years	Brazil	University hospital/academic departments	RYGB	Phase I Total—52 Surgery (Ob/OW) (before op.) = 26 Control (NW) = 26 Phase II Surgery (Ob/OW) (before and 12 and 24 months post op.) = 26	Mean 38 ± 2	79%	X	X	Self-reported oral health symptoms (i.e. Presence of gingivitis, periodontitis, bleeding gums when brushing and/or eating hard, dry foods; pain in the gums; and teeth with altered mobility), nausea, episodes of regurgitation, number of episodes of vomiting per day, tooth pain and dental hypersensitivity Oral Hygiene Habits (i.e. Frequency of tooth brushing, use of dental floss and fluoride, and dentist visits and their reasons) Salivary flow/secretion rate, buffering capacity and pH
Pataro et al. [29]	2012	Cross-sectional	-	Brazil	Specialized obesity treatment/bariatric surgery centre	RYGB (Fobi-Capella Technique)	Total = 345 Surgery (Ob/OW) (before op.) = 133 Surgery (Ob/OW) (≤ 6 months post op.) = 72 Surgery (Ob/OW) (> 6 months post op.) = 140	18–60 Mean 35 ± 9	86%		X	PlI, PPD, BOP, CAL, suppuration, tooth loss, periodontitis diagnosis
Marsicano et al. [35]	2012	Cross-sectional	-	Brazil	University hospital/academic departments	RYGB	Total = 102 Surgery (Ob/OW) = 52 Non-surgery (Ob/OW) = 50	Mean 38 ± 10	74%		X	DMFT, CPI and DWI Salivary flow/secretion rate
de Moura-Grec et al. [10]	2014	Phase I Cross-sectional Phase II Prospective longitudinal single-group observational	6 months	Brazil	Medical clinic(s)/centre(s)/hospital(s)	RYGB	Phase I Total = 110 Surgery (Ob/OW) (before op.) = 59 Control (NW) = 51 Phase II Surgery (Ob/OW) (before and 6 months post op.) = 59	Mean 39 ± 10	82%		X	Calculus, PPD, BOP, CAL and DWI Salivary flow/secretion rate, no. of teeth, no. of decayed teeth
Cardozo et al. [9]	2014	Prospective longitudinal single-group observational	6 months	Brazil	Specialized obesity treatment/bariatric surgery centre	RYGB	Total = 39 Surgery (Ob/OW) (before and 6 months post op.)	27–64 Mean 46 ± 10	97%	X	X	Self-reported oral health, access to dental care services, Toothbrushing frequency, flossing and dry mouth sensation ICDAS^c^ Salivary flow/secretion rate
Hashizume et al. [33]	2015	Prospective longitudinal single-group observational	6 months	Brazil	Specialized obesity treatment/bariatric surgery centre	RYGB	Total = 27 Surgery (Ob/OW) (before and 6 months post op.)	33–61 Mean 45 ± 8	96%		X	ICDAS, PlI and GI Salivary flow/secretion rate, pH, buffering capacity, microbial levels of mutans streptococci, *lactobacillus* spp., and *Candida albicans*
Sales-Peres et al. [11]	2015	Prospective longitudinal single-group observational	12 months	Brazil	Medical clinic(s)/centre(s)/hospital(s)	RYGB	Total = 50 Surgery (Ob/OW) (before and 6 and 12 months post op.)	Mean 39 ± 10	84%		X	GI, CI^c^, PPD, CAL and No. of teeth GCF^c^ for detection of *Porphyromonas gingivalis, Tannerella forsythia, Treponema denticola, and Prevotella intermedia*
Jaiswal et al. [25]	2015	Prospective longitudinal single-group interventional	6 months	India	University hospital/academic departments	(Not mentioned)	Total = 224 Surgery (Ob/OW) (before and 6 months post op.) (with periodontitis) (Intervention: non-surgical periodontal therapy and diet restriction)	18–64 Mean 49 ± 9	36%		X	PlI, GI, PPD, BOP, CAL
Knaś et al. [26]	2016	Prospective cohort	6 months	Poland	University hospital/academic departments	LSG^a^	Total = 80 Surgery (Ob/OW) (before and 6 months post op.) = 40 Control (NW) = 40	34–55	73%	X	X	Self-reported xerostomia (oral dryness) DMFT, SBI^c^, CAL and PPD Salivary flow/secretion rate, total antioxidant status (TAS), total oxidant status (TOS), oxidative stress index (OSI), superoxide dismutase 2 (SOD2), catalase (CAT) concentrations, specific activity of peroxidase (Px), uric acid (UA), malondialdehyde (MDA), and advanced glycation end products (AGE) as well as polyphenols (pPh) concentrations
Sales-Peres et al. [28]	2017	Prospective longitudinal single-group observational	12 months	Brazil	Medical clinic(s)/centre(s)/hospital(s)	RYGB	Total = 110 Surgery (Ob/OW) (before and 6 and 12 months post op.)	20–60 Mean 39 ± 10	88%		X	PPD, BOP, CAL
Weinberg et al. [23]	2018	Prospective longitudinal single-group observational	12 months	Israel	Medical clinic(s)/centre(s)/hospital(s)	LAP-Band, Sleeve Gastrectomy, BPD/DS, RYGB	Total = 50 Surgery (before and 6–18 months post op.) (Weight status not disclosed)	18–60 Mean 38 ± 12	52%	X	X	OHIP^c^−14 DMFT, PlI, calculus, PPD, BOP
Karlsson et al. [19]	2018	Case–control	-	Sweden	(Not mentioned)	(Not mentioned)	Total = 193 Surgery (Ob/OW) (> 1 year post op.) = 77 Non-surgery (Ob/OW) = 45 Non-surgery (NW) = 71	Surgery (Ob/OW): Mean 43 ± 11 Non-surgery (Ob/OW): Mean 44 ± 11 Non-surgery (NW): Mean 35 ± 10	91%	X		OHIP-S (Swedish version)
Aznar et al. [37]	2019	Cross-sectional	-	Brazil	University hospital/academic departments	RYGB	Total = 240 Surgery (Ob/OW) (≤ 24 months post op) = 60 Surgery (Ob/OW) (> 36 months post op) = 60 Non-surgery (Ob/OW) = 60 Nor-surgery (NW) = 60	Mean of all groups 36–39	80%		X	IDD^c^, tooth loss
Balogh et al. [24]	2020	Prospective Cohort	6–12 months	Hungary	University hospital/academic departments	RYGB	Total = 57 Surgery (Ob/OW) (before and 6–12 months post op.) = 17 Non-surgery (Ob/OW) = 18 Control (NW) = 22	18–58	58%		X	PPD, CAL, periodontitis diagnosis GCF for detection of *Actinomyces, Candida, Capnocytophaga, Eikenella, Fusobacterium, Granulicatella, Haemophilus, Lachnoanaerobaculum, Lactobacillus, Micrococcus, Neisseria, Prevotella, Rothia, Staphylococcus, Streptococcus,* and *Veillonella* genera
Taghat et al. [21]	2020	Cross-sectional	-	Sweden	Registry	RYGB	Total = 644 Surgery (> 2 years post op.) (Weight status not disclosed)	Mean 48 ± 12	75%	X		Self-reported oral health, number of teeth, chewing ability, oral health habits, oral symptoms (including tooth hypersensitivity), acid reflux episodes and vomiting episodes, and OHIP-49 (Swedish version)
Foratori-Junior et al. [74]	2020	Cross-sectional	-	Brazil	Medical clinic(s)/centre(s)/hospital(s)	RYGB	Total = 60 Surgery (Ob/OW) (≥ 12 months post op.) = 30 Non-surgery (Ob/OW) = 30	Surgery (Ob/OW): Mean 47 ± 11 Non-surgery (Ob/OW): Mean 38 ± 11	100%	X	X	Self-reported oral health and oral hygiene behaviours (i.e. frequency of toothbrushing and dental floss use) Tooth loss
Vargas et al. [32]	2020	Prospective cohort	6 months	Brazil	University hospital/academic departments	(Not mentioned)	Total = 31 Surgery (Ob/OW) (before and 6 months post op.) = 11 Control (NW) = 20	20–35	100%		X	Radiographic parameters: mandibular cortical index (MCI), Mentonian index (MI), panoramic mandibular index (MIP), bone level loss and trabecular pattern evaluation PI^c^
Yang et al. [39]	2021	Cross-sectional	-	Germany	University hospital/academic departments	LSG RYGB SADI-S^a^ BPD/DS	Total = 62 Surgery (Ob/OW) (≥ 3 months post op.) = 31 Non-surgery (Ob/OW) = 31	Surgery (Ob/OW): Mean 43 ± 10 Non-surgery (Ob/OW): Mean 38 ± 10	81%		X	BEWE
Alsuhaibani et al. [58]	2022	Cross-sectional	-	Saudi Arabia	Medical clinic(s)/centre(s)/hospital(s)	(Not mentioned)	Total = 250 Surgery (Ob/OW) (5–12 post op.)	30 to > 60	69%	X		Self-reported dental health (i.e. frequency of dental appointments, teeth brushing, use of fluoride toothpaste, use of fluoridated mouth rinse), postoperative oral symptoms (i.e. yellowing of the teeth, chipping, hypersensitivity)
Tinós et al. [31]	2022	Prospective cohort	12 months	Brazil	University hospital/academic departments	(Not mentioned)	Total = 89 Surgery (Ob/OW) (before and 1 year post op.) = 46 Non-surgery (Ob/OW) = 43	18–60	87%		X	ICDAS-II, BOP
Marquezin et al. [20]	2022	Controlled clinical	6 months	Brazil	Specialized obesity treatment/bariatric surgery centre	Vertical Roux-en-Y Gastroplasty	Total = 73 Surgery (Ob/OW) (diet program + before and 3 and 6 months post op.) = 39 Non-surgery (Ob/OW) (before and 3 and 6 months post diet program) = 34	19–59	82%	X	X	OHIP-14 (Brazilian version), XI^c^ (Portuguese version) DMFT, salivary flow/secretion rate, buffering capacity, total protein, alpha-amylase activity
van Leeuwen et al. [22]	2022	Cross-sectional	-	Netherlands	Medical clinic(s)/centre(s)/hospital(s)	Gastric bypass, sleeve gastrectomy	Total = 283 Surgery (Ob/OW) (1 year post op.) = 145 Non-surgery (Ob/OW) = 138	Surgery: Mean 52 ± 9 Non-surgery (Ob): Mean 45 ± 12	82%	X		OHIP-14
Čolak et al. [18]	2022	Randomized controlled clinical trial	3–6 months	Slovenia	University hospital/academic departments	OAGB^a^ RYGB LSG	Total = 30 Surgery (Ob/OW) (before and 3 and 6 months post op.) (with periodontitis) (Intervention: OHI + non-surgical periodontal therapy 4 weeks before surgery) = 15 Surgery (Ob/OW) (before and 3 and 6 months post op.) (with periodontitis) (Intervention: OHI + Low intensive supra-gingival plaque removal 4 weeks before surgery) = 15	Mean 51 ± 9	70%	X	X	Self-reported habits (i.e. smoking, alcohol consumption, regular weekly exercise, daily oral hygiene, and regular twice a year dental check-ups), OHIP-14 PlI, GBI^c^, PPD, BOP, recession, CAL
Taghat et al. [30]	2023	Prospective cohort	2 years	Sweden	Specialized obesity treatment/bariatric surgery centre	RYGB LSG	Total = 66 Surgery (Ob/OW) (before and 2 years post op.) = 40 Non-surgery (Ob/OW) (before and 2 years post diet program) = 26	Surgery (Ob/OW): Mean 29 ± 5 Non-surgery (Ob/OW): Mean 28 ± 5	100%	X	X	Self-reported toothbrushing, interdental cleaning, visits to the dentist the last 5 years, and reason for the most recent dental appointment ICDAS-II, plaque, BOP
Ribeiro et al. [36]	2023	Prospective cohort	6 months	Brazil	Medical clinic(s)/centre(s)/hospital(s)	Vertical Roux-en-Y Gastroplasty	Total = 40 Surgery (Ob/OW) (Diet program + before and 3 and 6 months post op.) = 20 Non-surgery (Ob/OW) (before and 3 and 6 months post diet program) = 20	Surgery (Ob/OW): Mean 32 ± 6 Non-surgery (Ob/OW): Mean 35 ± 7	75%		X	DMFT, CPI, salivary flow/secretion rate, buffering capacity, salivary cytokines concentrations of IL-6, IL-10, TNF-α, and IFNy and salivary microbiota
Marsk et al. [75]	2024	Retrospective cohort	10 years	Sweden	Registry	RYGB LSG	Total = 590,073 Surgery (Ob/OW) (0–10 years pot op.) = 53,643 Control (NW) = 536,430	Mean 41 ± 11	76%		X	Incidence of tooth extractions, restorative interventions, endodontic interventions and periodontal interventions
Kogawa et al. [41]	2024	Cross-sectional	-	Brazil	Medical clinic(s)/Centre(s)/hospital(s)	RYGB LSG	Total = 62 Surgery (Ob/OW) (≥ 1 year post op.) = 31 Control (NW) = 31	Median 60	87%	X	X	Self-reported xerostomia, salivary flow/secretion, buffering capacity and chromogranin A

^a^*BPD/DS*, biliopancreatic diversion with duodenal switch; *DS*, duodenal switch; *JI Bypass*, jejunoileal bypass; *LAP-Band*, laparoscopic adjustable gastric banding OR gastric band surgery; *LSG*, laparoscopic sleeve gastrectomy OR vertical sleeve gastrectomy OR gastric sleeve; *OAGB*, one anastomosis gastric bypass; *RYGB*, Roux-en-Y gastric bypass OR laparoscopic Roux-en-Y gastric bypass; *SADI-S*, single anastomosis duodeno-ileal bypass with sleeve gastrectomy; *SRVG*, silastic ring vertical gastroplasty

^b^*Ob*, obese; *OW*, overweight; *NW*, normal weight; *Post Op.*, post operation

^c^*BEWE*, Basic Erosive Wear Examination [76]; *BOP*, bleeding on probing; *CAL*, clinical attachment level/loss; *CI*, calculus index [77]; *CPI*, Community Periodontal Index; *DMFT*, number of decayed, missing and filled teeth; *DWI*, Dental Wear Index [78]; *GBI*, Gingival Bleeding Index; *GCF*, gingival crevicular fluid; *GI*, Gingival Index [79]; *HRQoL*, Health-Related Quality of Life; *ICDAS*, International Caries Detection and Assessment System [80]; *IDD*, Tooth Wear Index [81]; *OIDP*, Oral Impact affecting Daily Performance [82]; *OHIP*, Oral Health Impact Profile [83]; *PI*, Plaque Index [84]; *PlI*, Plaque Index [85]; *PPD*, Probing Pocket Depth; *SBI*, Sulcus Bleeding Index [86]; *XI*, Xerostomia Inventory[87]

### Study Design and Settings

Several designs were used, with a few papers reporting more than one design in the same study (Table 1). A single-group design, i.e. with no control group, was observed in 10 papers, while three clinical trials were included. Sixteen studies were conducted in non-academic clinical settings.

### Oral Health Indicators

Both subjective and objective oral health indicators were evaluated (Table 1). Different versions of the Oral Health Impact Profile (OHIP) were used to subjectively assess the oral health-related quality of life (OHRQoL) [18–23], in addition to other tools including questionnaires on self-reported oral hygiene practices, self-reported oral health and self-reported xerostomia..

Objective oral health variables were categorized into clinical, biologic and radiographic. The most common clinical periodontal variables were probing pocket depth (PPD) (10/33) [10, 11, 18, 23–29], bleeding on probing (BOP) (10/33) [10, 18, 23, 25–31], clinical attachment level (CAL) (9/33) [10, 11, 18, 24–29], dental plaque (8/33) [9, 18, 23, 25, 27, 29, 30, 32] and gingival bleeding (5/33) [11, 18, 25, 27, 33] (Table 1). Other periodontal parameters, composite indices or case definitions were also used including calculus deposits, gingival recession and the community periodontal index. Also, the number of decayed, missing and filled teeth (DMFT) (6/33) [20, 23, 26, 34–36] and the International Caries Detection and Assessment System (ICDAS) (4/33) were reported [9, 30, 31, 33]. Other frequent teeth-related variables included the Dental/Tooth Wear Index (DWI/DDI) (4/33) [10, 34, 35, 37] and Basic Erosive Wear Index (BEWE) (2/33) [38, 39] in addition to other variables.

Several papers evaluated saliva biological variables including salivary flow rate (11/33) [9, 10, 16, 20, 26, 33–36, 40, 41], buffering capacity (5/33) [20, 33, 36, 40, 41] and pH (3/33) [16, 33, 40] were evaluated (Table 1). Other variables including salivary cortisol, alpha-amylase activity, total protein levels, concentration of cytokines IL-6, IL-10, TNF-**α** and IFN-**γ** and chemistry of chloride and bicarbonate were also investigated [16, 20, 36, 42]. Salivary microbial levels of mutans streptococci, lactobacilli and *Candida albicans* [33] and microbiological findings from the gingival clavicular fluid (GCF) were reported in two papers [11, 24].

Radiographic parameters were observed using intra-oral periapical images and extra-oral orthopantomogram (OPG) [32] (Table 1).

### The Association Between Bariatric Surgery and Oral Health

#### A) The Association Between Bariatric Surgery and Subjective Oral Health Indicators

**Fig. 3:**
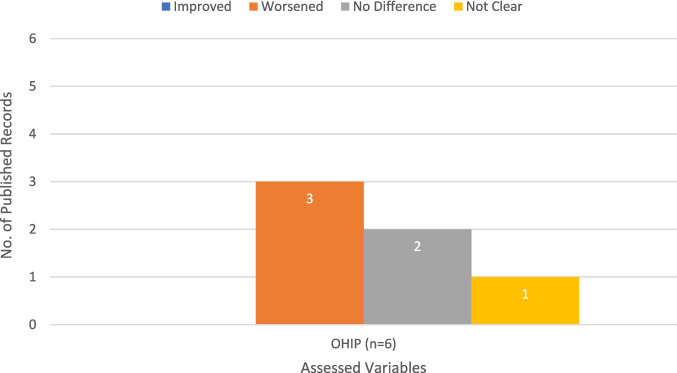
Bar chart showing the association between bariatric surgery and the subjective indicator using the Oral Health Impact Profile (OHIP) questionnaire as presented in the finally included original studies in the scoping review

Bariatric surgeries had a negative impact on OHIP scores [19–21] (Fig. 3). The domain ‘functional limitation’ in particular was lower compared to pre-operative obesity status [22].

#### B) The Association Between Bariatric Surgery and Objective Oral Health Indicators

Regarding periodontal indicators, a few studies reported notable improvements in plaque [25, 27, 32] and gingival bleeding [25, 27] following BS, but worsening in PPD [10, 11, 29], BOP [10, 28–31] and CAL [10, 11, 29] was also reported (Table 2). Five systematic reviews concurred the worsening in periodontal parameters, particularly in CAL, 6 months following BS, with a notable improvement in plaque scores [43–47].

**Table 2:** The association between bariatric surgery and common objective oral health findings as presented in the finally included original studies in the scoping review

Objective indicator category Indicator sub-category Specific indicator (No. of reporting studies)	Changes following bariatric surgery	No. of studies	References
**Clinical indicators**			
**Periodontal**			
Plaque (8/33)	Improved	3	[25, 27, 32]
	Worsened	2	[29, 30]
	No difference	2	[18, 23]
	Not clearly reported	1	[33]
GB^a^ (5/33)	Improved	2	[25, 27]
	Worsened	1	[11]
	No difference	-	-
	Not clearly reported	2	[18, 33]
PPD^a^ (10/33)	Improved	2	[18, 27]
	Worsened	3	[10, 11, 29]
	No difference	5	[23–26, 28]
	Not clearly reported	-	-
BOP^a^ (10/33)	Improved	3	[18, 25, 27]
	Worsened	5	[10, 28–31]
	No difference	2	[23, 26]
	Not clearly reported	-	-
CAL^a^ (9/33)	Improved	1	[27]
	Worsened	3	[10, 11, 29]
	No difference	5	[18, 24–26, 28]
	Not clearly reported	-	-
**Cariological**			
DMFT^a^ (6/33)	Improved	-	
	Worsened	4	[20, 23, 34, 36]
	No difference	2	[26, 35]
	Not clearly reported	-	-
ICDAS^a^ (4/33)	Improved	-	-
	Worsened	2	[30, 31]
	No difference	-	-
	Not clearly reported	2	[9, 33]
**Tooth wear**			
DWI^a^ (4/33)	Improved	-	-
	Worsened	3	[10, 34, 37]
	No difference	1	[35]
	Not clearly reported	-	-
BEWE^a^ (2/33)	Improved	-	-
	Worsened	1	[38]
	No difference	1	[39]
	Not clearly reported	-	-
**Biologic indicators**			
Salivary SR^a^ (11/33)	Improved	3	[10, 34, 40]
	Worsened	2	[16, 26]
	No difference	5	[20, 33, 35, 36, 41]
	Not clearly reported	1	[9]
Salivary BC^a^ (5/33)	Improved	-	-
	Worsened	3	[20, 40, 41]
	No difference	2	[33, 36]
	Not clearly reported	-	-
Salivary pH (3/33)	Improved	-	-
	Worsened	1	[16]
	No difference	1	[33]
	Not clearly reported	1	[40]

^a^*BEWE*, Basic Erosive Wear Examination; *BOP*, bleeding on probing; *CAL*, clinical attachment level/loss; *DMFT*, number of decayed, missing and filled teeth; *DWI*, Dental Wear Index; *GB*, gingival bleeding; *ICDAS*, International Caries Detection and Assessment System; *PPD*, probing pocket depth; *Salivary BC*, salivary buffer capacity; *Salivary SR*, salivary flow/secretion rate

Several studies showed a negative influence for BS on DMFT [20, 23, 34, 36] and ICDAS [30, 31] (Table 2). One systematic review observed a greater risk for developing caries among individuals undergoing BS [48], while another review failed to observe such a relation [45]. Several studies found that BS negatively influenced the DWI/DDI [10, 34, 37] (Table 2). Three systematic reviews confirmed the association between BS and dental wear and/or erosion [45, 47, 49].

With regard to biologic indicators, no change in salivary flow was observed [20, 33, 35, 36, 41] (Table 2). Two systematic reviews confirmed the absent association between BS and salivary secretion [45, 50]. Conversely, saliva buffering capacity [20, 40, 41] and pH [16] were negatively influenced by BS. Similarly, salivary cortisol and TNFα were unchanged following BS, but concentrations of IL-8, IL-10 and IFN-γ increased [36, 42]. On the other hand, salivary alpha amylase activate, total protein [20] and carbonate levels [16] decreased following BS. Salivary mutans streptococci, lactobacilli and *Candida albicans* increased [33] and *Candida albicans* and non-albicans appeared significantly in GCF after BS [24].

Radiographically, the mandibular cortical index, panoramic mandibular index, alveolar bone level and trabecular pattern were negatively influenced by BS, whereas only the Mentonian Index improved [32].

## Discussion

This review aimed at mapping the literature on the oral health of patients treated with BS. Despite the allocation of nine systematic reviews up to the data extraction date, this scoping review provides a broader and connected view of oral health aspects of such individuals and therefore facilitates meaningful recommendations for oral healthcare.

The OHRQoL decreased after bariatric surgery in more than half of the reporting studies [19–21]. Particularly, the domain ‘functional limitation’ was negatively affected [22]. This outcome was expected as major changes in lifestyle, and dietary habits are anticipated following BS [51]. Moreover, musculoskeletal, nutritional and psychological side effects of BS [52] could impact the quality of an individual’s life.

Concerning periodontal status following BS, results diverged between ‘got worse’, ‘stayed the same’ or ‘improved’ [28]. These conflicting findings may be explained by the lack of harmonization in study objectives, design and recruitment. It is noteworthy that the worsening of existing periodontal disease was limited to the 6 months after BS and could be improved by non-surgical periodontal therapy [18]. Moreover, a reported increase in an established periodontal pathogen, *Porphyromonas gingivalis* 6 months after BS, may contribute to the observed periodontal changes in the post-surgical period [11]. This factor alone, though, cannot explain such changes according to the ecological plaque hypothesis and calls for further research [53].

An increase in dental caries in BS individuals has been reported [34]. A systematic review confirmed the increased caries risk in such individuals compared to those who had not had BS [48]. Multiple factors including the increased number of daily meals following BS might play a role in the increased risk [36]. Higher levels of mutans streptococci 6 months after BS have also been reported [33]. In addition, salivary pH and buffering capacity were negatively associated with BS [16, 20, 40, 41]. A case series reported that most BS patients had poor oral hygiene and hyposalivation, despite conflicting reports on plaque and salivation [54]. Such findings highlight the importance of post-operative follow-up [55]. In addition, these findings suggest the need for more innovative strategies for better patient education and adherence [56].

An association between tooth wear, especially dental erosion, and BS was also reported [39]. This association is understandable, as gastroesophageal reflux disease (GERD) and vomiting are common complications after BS [57, 58]. This points back to the importance of regular follow up and monitoring by an oral healthcare professional post-surgery for dietary advice.

An overall reduction in the inflammatory status was observed after BS [59]. The current review, on the other hand, highlights that a number of inflammatory markers increased in saliva after surgery, while others decreased [36]. This interesting finding may point to the different roles inflammatory markers have, as some are designated as pro-inflammatory, while others as anti-inflammatory [60]. Furthermore, a persistent low-grade inflammation has been reported after BS, supporting the hypothesis of the adipose tissues suffering from a so-called obesity imprint even after weight loss [61, 62].

Poor dietary habits are established contributors to the development of obesity, especially over consumption of high-calorie and palatable foods rather than bitter-tasting foods and fibrous vegetables [63]. Since dietary habits are substantially influenced by taste perception, analysing saliva has been particularly useful [64]. Specifically, salivary carbonic anhydrase VI and other proteins have been linked to bitter taste [65]. Alpha amylase, on the other hand, has long been connected to the perception of starch [66]. A decrease in salivary alpha amylase activate, carbonate and total protein levels following BS has been observed [20]. Such changes coincide with the desired weight loss and may be a useful parameter to monitor during follow-up visits.

A decrease in salivary cortisol (i.e. the stress hormone) among women with morbid obesity has been described [67]. Stressful life events may be associated with uncontrollable eating habits such the binge eating disorder (BED), ultimately contributing to the development of obesity [68]. However, no significant change in salivary cortisol following BS was found, regardless of the time of the day of collection [42]. Cortisol, which can be detected in body fluids including serum, urine and saliva, normally proceeds in pulses and is associated with rhythms of sleep and wakefulness [69]. Accordingly, single cortisol assessments may strongly be impacted by acute measurement aspects including the time of the day, day of the week and sampling distress [70]. Furthermore, widespread use of salivary cortisol collection is hampered by poorly standardized assays, the lack of validated reference ranges, variable sampling techniques and risk of sample contamination [71].

Radiographs reveal negative changes in bone following BS [32], and these changes have been associated with changes in bone metabolism and progressive bone loss [72]. Such findings suggest the need for monitoring the overall bone status upon following up of BS patients and suggests further long-term research to evaluate the potential impact of BS on tooth-supporting alveolar bone.

## Limitations and Strengths

The included studies varied considerably in aim, design, populations and studied outcomes. Furthermore, the quality of the included studies was not specifically evaluated, although visibly different between reports. However, it is necessary to consider that the current scoping review aims to provide a broader connection to the available literature and allocate areas designated as knowledge gaps for future research. An important strength of this review is its was the comprehensive methodology used while conducting the search, marking the present paper a possible reference for future work, in an increasingly growing area of interest.

## Conclusions

Within the limitations of this scoping review, it is evident that the literature on oral health in patients who have undergone BS is continuously growing. Several papers, with various study designs, inclusion criteria and settings, suggested an improvement in subjective and objective oral health parameters following weight loss surgery. However, adverse oral health effects of surgery have been reported. Confirmation of these findings will require further focused research with standardized study designs and outcomes and long-term evaluations. Nevertheless, the available literature can be used to develop clinical recommendations for the follow-up of BS patients and the specific oral health aspects that require long-term monitoring can be based on the available literature.

## Supplementary Information

Below is the link to the electronic supplementary material.

Supplementary file1 (DOCX 17 KB)

Supplementary file2 (DOCX 18 KB)

Supplementary file3 (DOCX 18 KB)

Supplementary file4 (DOCX 2440 KB)

Supplementary file5 (DOCX 144 KB)

## Data Availability

No datasets were generated or analysed during the current study.
