# Supplementary material for: Oral Health in Individuals After Bariatric Surgery: A Systematic Scoping Review
Source: Obes Surg. 2025 Mar 19;35(5):1878–99. doi: 10.1007/s11695-025-07793-w (PMC12065770; doi:10.1007/s11695-025-07793-w)
Supplement: Supplementary file 3 — Supplementary file3 (DOCX 18 KB) [file 11695_2025_7793_MOESM3_ESM.docx]

Appendix 3: Keywords and Search Strategy (3^rd^ search)

**Updated search (3^rd^ search) 2024-11-11**Number of results in total 2070
After deduplication 1105

After deduplication against 2^nd^ search 65

**PubMed 2024-11-11**

#1
bariatric Surgery[mh] OR gastrectomy[mh] OR Obesity/surgery[mh] OR gastroenterostomy[mh] OR bariatric surgery[tiab] OR bariatric surgical[tiab] OR bariatric operation*[tiab] OR gastric bypass[tiab] OR stomach bypass[tiab] OR obesity surgery[tiab] OR gastroplasty[tiab] OR gastroplasties[tiab] OR gastric band*[tiab] OR RYGB[tiab] OR Roux-en-Y[tiab] OR LAGB[tiab] OR LRYGB[tiab] OR gastroenterostomy[tiab] OR gastroenterostomies[tiab] OR gastrectomy[tiab] OR weight loss surgery[tiab] OR gastric sleeve[tiab] OR stomach sleeve[tiab] OR endoluminal sleeve[tiab] OR jejunoileal bypass[tiab] OR biliopancreatic diversion[tiab] OR scopinaro[tiab] OR duodenal switch[tiab] OR “stomach band”[tiab] OR Mason procedure[tiab] OR stomach stapling[tiab] OR intragastric balloon*[tiab] OR stomach fold*[tiab] OR gastric imbrication[tiab] OR gastric plication*[tiab]
 🡪 104,126

#2
Oral health[mh] OR salivation[mh] OR periodontal diseases[mh] OR biofilms[mh] OR tooth diseases[mh] OR oral health[tiab] OR caries[tiab] OR saliva*[tiab] OR “dental erosion”[tw] OR tooth erosion[tiab] OR “cariogenic bacteria”[tw] OR biofilm*[tw] OR periodontitis[tiab] OR gingivitis[tiab] OR dental[tiab] OR dentition[tiab] OR tooth[tiab] OR teeth[tiab] OR oral condition*[tiab] OR oral disease*[tiab] OR oral hygiene[tiab] OR mouth[tiab] OR oral habit*[tiab] OR oral pH[tiab] OR oral acid[tiab] OR abfraction[tiab] OR bruxism[tiab] OR clenching[tiab] OR oral flora[tiab] OR gingiva*[tiab] OR periodont*[tiab] OR pocket depth*[tiab] OR peri-implant*[tiab] OR gum[tiab] OR oral muco*[tiab] OR taste[tiab] OR Tongue[tiab] OR Mandible[tiab] OR Maxilla[tiab] OR Jaw[tiab] OR Alveolar Bone[tiab]

- 1,022,722

**#1 AND #2 🡪 659**

## Scopus 2024-11-11

## #1 TITLE-ABS ( "bariatric surgery" OR "bariatric surgical" OR "bariatric operation*" OR "gastric bypass" OR "stomach bypass" OR "obesity surgery" OR gastroplasty OR gastroplasties OR "gastric band*" OR rygb OR "Roux-en-Y" OR lagb OR lrygb OR gastroenterostomy OR gastroenterostomies OR gastrectomy OR "weight loss surgery" OR "gastric sleeve" OR "stomach sleeve" OR "endoluminal sleeve" OR "jejunoileal bypass" OR "biliopancreatic diversion" OR scopinaro OR "duodenal switch" OR "stomach band" OR "Mason procedure" OR "stomach stapling" OR "intragastric balloon*" OR "stomach fold*" OR "gastric imbrication" OR "gastric plication*" ) 🡪 84,797

## #2 TITLE-ABS ( “oral health”  OR  caries  OR  saliva*  OR  “dental erosion”  OR  “tooth erosion”  OR  “cariogenic bacteria”  OR  biofilm*  OR  periodontitis  OR  gingivitis  OR  dental  OR  dentition  OR  tooth  OR  teeth  OR  “oral condition*”  OR  “oral disease*”  OR  “oral hygiene”  OR  mouth OR “oral habit*”  OR  “oral pH”  OR  “oral acid”  OR  abfraction  OR  bruxism  OR  clenching  OR  “oral flora”  OR  gingiva*  OR  periodont*  OR  “pocket depth*”  OR  “peri-implant*”  OR  gum  OR  “oral muco*”  OR  taste  OR  tongue  OR  mandible  OR  maxilla  OR  jaw  OR  “alveolar bone” ) 🡪 1,381,572

**#1 AND #2 🡪 604**

**Web of Science 2024-11-11**

#1
TS=(”bariatric surgery” OR ”bariatric surgical” OR ”bariatric operation*” OR ”gastric bypass” OR ”stomach bypass” OR ”obesity surgery” OR gastroplasty OR gastroplasties OR ”gastric band*” OR rygb OR ”Roux-en-Y” OR lagb OR lrygb OR gastroenterostomy OR gastroenterostomies OR gastrectomy OR ”weight loss surgery” OR ”gastric sleeve” OR ”stomach sleeve” OR ”endoluminal sleeve” OR ”jejunoileal bypass” OR ”biliopancreatic diversion” OR scopinaro OR ”duodenal switch” OR ”stomach band” OR ”Mason procedure” OR”stomach stapling” OR ”intragastric balloon*” OR ”stomach fold*” OR ”gastric imbrication” OR ”gastric plication*”)

- 93,411

#2
TS=(”oral health” OR caries OR saliva* OR ”dental erosion” OR ”tooth erosion” OR ”cariogenic bacteria” OR biofilm* OR periodontitis OR gingivitis OR dental OR dentition OR tooth OR teeth OR ”oral condition*” OR ”oral disease*” OR ”oral hygiene” OR mouth OR TITLE-ABS-KEY ”oral habit*” OR ”oral pH” OR ”oral acid” OR abfraction OR bruxism OR clenching OR ”oral flora” OR gingiva* OR periodont* OR ”pocket depth*” OR ”peri-implant*” OR gum OR ”oral muco*” OR taste OR tongue OR mandible OR maxilla OR jaw OR ”alveolar bone”)

- 1,090,775

**#1 AND #2 🡪 767**
