# Supplementary material for: Oral Health in Individuals After Bariatric Surgery: A Systematic Scoping Review
Source: Obes Surg. 2025 Mar 19;35(5):1878–99. doi: 10.1007/s11695-025-07793-w (PMC12065770; doi:10.1007/s11695-025-07793-w)
Supplement: Supplementary file 4 — Supplementary file4 (DOCX 2440 KB) [file 11695_2025_7793_MOESM4_ESM.docx]

Appendix 4: Original studies included in the scoping review (n=33).

| Authors | Year | Study Title | Study Objective(s) | Study Type | Country^a^ | Settings^b^ | Study Duration | Type of Bariatric Surgery^c^ | Inclusion criteria  (Test group) | Inclusion criteria  (Control group) | Number of Participants^d^ | Age (years) | Sex  (F%) | Studied Variables Category | | Variables Sub-category | Studied Outcome Variable(s)^e^ | Key findings related to scoping review question |
| --- | --- | --- | --- | --- | --- | --- | --- | --- | --- | --- | --- | --- | --- | --- | --- | --- | --- | --- |
|  |  |  |  |  |  |  |  |  |  |  |  |  |  | Subjective | Objective |  |  |  |
| Greenway and Greenway | 2000 | Root Surface Caries: A Complication of the Jejunoileal Bypass | To document the association of root surface caries with the Jejunoileal Bypass operation, explore the mechanism responsible for this association and suggest potential methods to prevent or minimize the problem. | Phase I Cross-Sectional  Phase II Cross-Sectional | USA | (Not mentioned) | - | JI Bypass | Weight more than 136.4kg, age less than 30 years, having no pre-existing disease | Weight 90-110 kg, age 20-40 years, having no pre-existing disease | Phase I Total = 23 Surgery (Ob/OW) (Average 10 years post op.) = 18 Control (NW) = 5  Phase II Total = 8 Surgery (Ob/OW) (25-30 years post op.) = 4 Control (NW) = 4 | Phase I Surgery: <30 (Mean 25) Control: 24-40  Phase II Surgery: 36-56 (Mean 48±9) Control: 25-54 (Mean 42±13) | (Not mentioned) | X | X | Subjective | Phase I Surgery group only, before and after surgery: Self-reported root surface caries. Surgery and control groups: Salivary chloride, bicarbonate and pH.  Phase II Surgery and control groups: Stimulated salivary flow/secretion rate. | After JI bypass, the incidence of root surface caries per year increased. The increase in root surface caries was associated with a decrease in salivary flow-rate, pH and salivary buffering capacity. |
| Heling et al. | 2006 | Dental Complications Following Gastric Restrictive Bariatric Surgery | To investigate the self-assessment of bariatric patients regarding their dental health status. | Cross-sectional | Israel | Medical Clinic(s) / Center(s) / Hospital(s) | - | SRVG Lap-Band.SRVG | Underwent bariatric surgery 1 to 10 years prior to the survey | - | Total = 113 Surgery (1-10 years post op.)  (Weight status not disclosed) | Mean 40±10 | 75% | X |  | Subjective | Self-reported oral hygiene practices, frequency of visits to the dentist, hypersensitivity of the teeth, dental treatment and sense of taste. | Lack of adequate previous referral and the attitude of the health authorities are a cause for concern and call for improved and increased oral health promotional activity. |
| Marsicano et al. | 2011 | Interfaces between bariatric surgery and oral health. A longitudinal survey | To verify oral alterations in patients before and after bariatric surgery, identify the occurrence of dental caries, periodontal diseases and dental wear, and to correlate oral health with quality of life in these patients. | Prospective Longitudinal Single-Group Observational | Brazil | University Hospital / Academic Departments | 6 months | RYGB | Underwent RYGB at Clinical Hospital of the Faculty of Medicine, Ribeirão Preto, University São Paulo | - | Total = 54 Surgery (Ob/OW) (Before and 3- and 6-months post op.) | Mean 41±10 | 81% | X | X | Subjective  Clinical  Biologic | OIDP. DMFT, CPI and DWI. Salivary flow/secretion rate. | After bariatric surgery patients showed an increase in the occurrence of dental caries and in the severity of tooth wear, however these alterations in oral health status did not influence the quality of life, probably as a result of the significant improvement in the general health of these patients. Saliva flow was reduced in patients before surgery and increased after RYGB. presence of periodontal pockets in 50%, 58% and 50% of patients before, 3 and 6 months after surgical treatment respectively. |
| Valentine et al. | 2011 | Salivary Cortisol Increases After Bariatric Surgery in Women | To prospectively evaluate cortisol dynamics and health-related quality of life as measures of stress among obese women before and at long-term follow-up after bariatric surgery. | Prospective Longitudinal Single-Group Observational | USA | Medical Clinic(s) / Center(s) / Hospital(s) | 12 months | LSG Lap-Band RYGB DS | Obese participants not using exogenous glucocorticoids | - | Total = 24 Surgery (Ob/OW) (Before and 6- and 12-months post op.) | Mean 49±8 | 100% | X | X | Subjective  Biologic | HRQoL questionnaire using the Medical Outcomes Study Health Survey (Short-Form 12, version 2) and salivary cortisol. | Morning cortisol levels increase, but remain within normal ranges, 6–12 months after bariatric surgery. |
| Alves et al. | 2012 | Tooth Wear in Patients Submitted to Bariatric Surgery | To determine prevalence of tooth wear as well as the level of risk in a sample of bariatric patients from a Brazilian public hospital. | Cross-sectional | Brazil | University Hospital / Academic Departments | - | (Not mentioned) | Bariatric group: Patients who had been submitted to bariatric surgery at least 6 months previously.  Obese group: Patients on the waiting list for this surgery | Patients from other sectors of the same hospital who were waiting for an ambulatory medical care | Total = 125 Surgery (Ob/OW) (≥6 months pot op.) = 41 Non-surgery (Ob/OW) = 42 Control (NW) = 42 | Surgery Mean 43±9  Non-surgery Mean 37±10  Control Mean 33±10 | 86% |  | X | Clinical | BEWE | Bariatric patients showed higher prevalence and a statistically significant level of risk with regard to non-carious dental lesions when compared with the other patients, followed by the obese and control groups. Reflux and vomiting did not seem to influence non-carious dental lesions positively. |
| Lakkis et al. | 2012 | Response to Periodontal Therapy in Patients Who Had Weight Loss After Bariatric Surgery and Obese Counterparts: A Pilot Study | To assess whether significant weight loss by BS improves the response to non-surgical periodontal therapy. | Controlled Clinical | USA | University Hospital / Academic Departments | 4-6 weeks | (Not mentioned) | Age ≥18 years, diagnosis of chronic periodontitis (classified as a mean clinical attachment loss of ≥2 mm), ≥20 teeth present, BMI >30 kg/m2, loss of ≥40% excess weight after bariatric surgery. | - | Total = 30 Surgery (Ob/OW) (≥6 months pot op.) = 15 Non-surgery (Ob/OW) = 15  (Intervention: Non-Surgical Periodontal Therapy) | Mean 47±11 | 63% |  | X | Clinical | PlI, GI, PPD, BOP and CAL | Improved response to non-surgical periodontal therapy in obese patients who lost significant weight after bariatric surgery compared with obese patients who did not have such a surgery. |
| Netto et al. | 2012 | Influence of Roux-en-Y Gastric Bypass Surgery on Vitamin C, Myeloperoxidase, and Oral Clinical Manifestations: A 2-Year Follow-Up Study | To investigate the influence of RYGB surgery on vitamin C and MPO levels and disease development in the oral cavity. | Phase I Cross-Sectional  Phase II Prospective Longitudinal Single-Group Observational | Brazil | University Hospital / Academic Departments | 2 years | RYGB | Age 20–59 years, BMI ≥40 kg/m2 or ≥35 kg/m2 with a comorbidity | Normal weight as defined by WHO | Phase I Total - 52 Surgery (Ob/OW) (Before op.) = 26 Control (NW) = 26  Phase II Surgery (Ob/OW) (Before and 12- and 24- months post op.) = 26 | Mean 38±2 | 79% | X | X | Subjective  Biologic | Self-reported oral health symptoms (i.e. Presence of gingivitis, periodontitis, bleeding gums when brushing and/or eating hard, dry foods; pain in the gums; and teeth with altered mobility), nausea, episodes of regurgitation, number of episodes of vomiting per day, tooth pain and dental hypersensitivity,  Oral Hygiene Habits (i.e. Frequency of tooth brushing, use of dental floss and fluoride, and dentist visits and their reasons). Salivary flow/secretion rate, buffering capacity and pH. | Vitamin C deficiency and increased vomiting after RYGB for morbid obesity may contribute to increased periodontal disease. At 24 months after surgery, saliva flow was significantly higher compared with the basal period. The basal-buffering capacity of saliva in the BG was above the reference value. At 12 months, this parameter was significantly reduced. |
| Pataro et al. | 2012 | Influence of Obesity and Bariatric Surgery on the Periodontal Condition | To determine and compare the periodontal condition of: 1) individuals recommended for bariatric surgery; 2) those having undergone bariatric surgery within a postoperative period of ≤ months; and 3) those having undergone bariatric surgery >6 months before the study. Moreover, the influence of biologic, demographic, and behavioral risk variables in this possible association were also analyzed. | Cross-sectional | Brazil | Specialized Obesity Treatment / Bariatric Surgery Center | - | RYGB (Fobi-Capella Technique) | Obese patients in preoperative or postoperative periods for bariatric surgery who were being monitored in a reference center for the treatment of obesity in Belo Horizonte, Minas Gerais, Brazil  and who had undergone a complete periodontal examination between March 2008 and March 2010. | - | Total = 345 Surgery (Ob/OW) (Before op.) = 133 Surgery (Ob/OW) (≤6 months post op.) = 72 Surgery (Ob/OW) (>6 months post op.) = 140 | 18-60 Mean 35±9 | 86% |  | X | Clinical | PlI, PPD, BOP, CAL, supporation, tooth loss, periodontitis diagnosis. | Different times of the bariatric surgery, showing a high prevalence of periodontitis in both preoperative and postoperative follow-up. |
| Marsicano et al. | 2012 | Evaluation of oral health status and salivary flow rate in obese patients after bariatric surgery | To compare the prevalence of dental caries, periodontal diseases and dental wear in bariatric patients and morbidly obese patients and to correlate the conditions of oral health with saliva flow. | Cross-sectional | Brazil | University Hospital / Academic Departments | - | RYGB | Underwent RYGB surgery | Severely obese patients selected for bariatric surgery | Total = 102 Surgery (Ob/OW) = 52 Non-surgery (Ob/OW) = 50 | Mean 38±10 | 74% |  | X | Clinical  Biologic | DMFT, CPI and DWI. Salivary flow/secretion rate. | Bariatric patients showed a prevalence of oral diseases similar to that of obese patients, nevertheless there was a more prevalent condition of periodontal pockets in bariatric patients |
| de Moura-Grec et al. | 2014 | Impact of bariatric surgery on oral health conditions: 6-months cohort study | To verify periodontal conditions and changes in dental wear after bariatric surgery. | Phase I Cross-Sectional  Phase II Prospective Longitudinal Single-Group Observational | Brazil | Medical Clinic(s) / Center(s) / Hospital(s) | 6 months | RYGB | Morbidly obese patients recommended for RYGB | Normal BMI, without systemic disease, similar age range, living in the State of Sao Paulo, users of the system of public health | Phase I Total = 110 Surgery (Ob/OW) (Before op.) = 59 Control (NW) = 51  Phase II Surgery (Ob/OW) (Before and 6 months post op.) = 59 | Mean 39±10 | 82% |  | X | Clinical  Biologic | Calculus, PPD, BOP, CAL and DWI. Salivary flow/secretion rate, no. of teeth, no. of decayed teeth. | Negative impact on oral health conditions, reflected by the increase in periodontal disease (PPD, CAL, BOP) and dental wear (DWI), while salivary flow slightly increased. |
| Cardozo et al. | 2014 | Impact of Bariatric Surgery on the Oral Health of Patients with Morbid Obesity | To investigate the association between bariatric surgery and changes in the oral health status of patients with morbid obesity. | Prospective Longitudinal Single-Group Observational | Brazil | Specialized Obesity Treatment / Bariatric Surgery Center | 6 months | RYGB | 18 years or older, having the cognitive ability to respond to the questionnaire and physical ability to travel to the location of the examination, being hospitalized for bariatric surgery on the day after the oral examination. | - | Total = 39 Surgery (Ob/OW) (Before and 6 months post op.) | 27-64 Mean 46±10 | 97% | X | X | Subjective  Clinical  Biologic | Self-reported oral health, access to dental care services, Toothbrushing frequency, flossing and dry mouth sensation. ICDAS. Salivary flow/secretion rate. | There was a decrease in the sensation of dry mouth and an increased stimulated salivary flow |
| Hashizume et al. | 2015 | Impact of Bariatric Surgery on the Saliva of Patients with Morbid Obesity | To evaluate the salivary conditions of morbidly obese patients prior to bariatric surgery and 6 months after surgery. | Prospective Longitudinal Single-Group Observational | Brazil | Specialized Obesity Treatment / Bariatric Surgery Center | 6 months | RYGB | 18 years or older, having the cognitive ability to respond to the questionnaire and physical ability to travel to the location of the examination, being hospitalized for bariatric surgery on the day after the oral examination. | - | Total = 27 Surgery (Ob/OW) (Before and 6 months post op.) | 33-61 Mean 45±8 | 96% |  | X | Clinical  Biologic | ICDAS, PlI and GI. Salivary flow/secretion rate, pH, buffering capacity, microbial levels of mutans streptococci, lactobacillus spp., and Candida albicans. | There were no statistical differences, before or after surgery, for stimulated salivary flow rate, salivary pH, buffering capacity of saliva, levels of Lactobacillus spp., and levels of Candida albicans. However, there was significantly increase in the levels of mutans streptococci in saliva of patients with morbid obesity after 6 months of bariatric surgery. |
| Sales-Peres et al. | 2015 | Periodontal status and pathogenic bacteria after gastric bypass: a cohort study | To investigate whether significant weight loss by Roux-en-Y gastric bypass would decrease the presence of periodonto-phatogenic bacteria and periodontal diseases in morbid obese patients in a 12-month follow up | Prospective Longitudinal Single-Group Observational | Brazil | Medical Clinic(s) / Center(s) / Hospital(s) | 12 months | RYGB | Morbid obese patient indicated for bariatric surgery and treated at the Public Health System of Brazil | - | Total = 50 Surgery (Ob/OW) (Before and 6- and 12-months post op.) | Mean 39±10 | 84% |  | X | Clinical  Biologic | GI, CI, PPD, CAL and No. of teeth. GCF for detection of Porphyromonas gingivalis, Tannerella forsythia, Treponema denticola, and Prevotella intermedia. | The periodontal disease seems to have increased in severity after gastric bypass surgery (PPD, CAL and GI worsened in 6 months), which may increase the risk of cardiovascular disease. The periodontopathogenic bacteria quantification revealed alterations during three periods (pre-operative, 6 months and 12 months) and P. gingivalis was the bacteria that influenced the severity increase in periodontal disease. |
| Jaiswal et al. | 2015 | Impact of Bariatric Surgery and Diet Modification on Periodontal Status: A Six Month Cohort Study | To verify alterations in periodontal status in patients before and after bariatric surgery, and to evaluate if a correlation exists between diet modification, oral prophylaxsis and periodontal status of these patients. | Prospective Longitudinal Single-Group Interventional | India | University Hospital / Academic Departments | 6 months | (not mentioned) | Age >18 years, Diagnosis of periodontitis classified as mean clinical attachment loss of >2mm, More than 20 teeth present, BMI > 30kg/m2 | - | Total = 224 Surgery (Ob/OW) (Before and 6 months post op.) (With Periodontitis)  (Intervention: Non-Surgical Periodontal Therapy and diet restriction) | 18-64 Mean 49±9 | 36% |  | X | Clinical | PlI, GI, PPD, BOP, CAL. | In spite of not performing any aggressive periodontal treatment the percentage of bleeding sites, gingival index and plaque index were significantly reduced post-surgery as compared to the pre-surgical recording, the difference of which was found to be statistically significant. While CAL and PPD values were similar at baseline and after bariatric surgery. |
| Knaś et al. | 2015 | Impact of morbid obesity and bariatric surgery on antioxidant/oxidant balance of the unstimulated and stimulated human saliva | To evaluate the influence of morbid obesity and bariatric surgery on antioxidant/ oxidant homeostasis of the unstimulated and stimulated human saliva. | Prospective Cohort | Poland | University Hospital / Academic Departments | 6 months | LSG | Morbid obese individuals (BMI > 40) from 1st Department of General Surgery and Endocrinology from among bariatric surgery patients. | 18.5 ≤ BMI ≥ 24.5, not smokers, did not have any illness | Total = 80 Surgery (Ob/OW) (Before and 6 months post op.) = 40 Control (NW) = 40 | 34-55 | 73% | X | X | Subjective  Clinical  Biologic | Self-reported xerostomia (oral dryness). DMFT, SBI, CAL and PPD. Salivary flow/secretion rate, total antioxidant status (TAS), total oxidant status (TOS), oxidative stress index (OSI), superoxide dismutase 2 (SOD2), catalase (CAT) concentrations, specific activity of peroxidase (Px), uric acid (UA), malondialdehyde (MDA), and advanced glycation end products (AGE) as well as polyphenols (pPh) concentrations. | Bariatric surgery restored only unstimulated salivary flow to normal values. Disturbances in oxidant/antioxidant homeostasis can be observed in unstimulated saliva and stimulated saliva of morbid obese patients before and after treatment. DMFT, SBI index, PPD, and CAL values were similar between groups. |
| Sales-Peres et al. | 2017 | Weight loss after bariatric surgery and periodontal changes: a 12-month prospective study | To explore whether weight loss after bariatric surgery was associated with changes in periodontal measures over 12 months. | Prospective Longitudinal Single-Group Observational | Brazil | Medical Clinic(s) / Center(s) / Hospital(s) | 12 months | RYGB | Morbidly obese patients (BMI>40 kg/m2 or ≥35 kg/m2 with comorbid conditions) recruited from the patient pool receiving bariatric surgery (laparoscopic Roux-en-Y gastric bypass) in 2 public hospitals in São Paulo, Brazil, between April 2011 and March 2013. | - | Total = 110 Surgery (Ob/OW) (Before and 6- and 12-months post op.) | 20-60 Mean 39±10 | 88% |  | X | Clinical | PPD, BOP, CAL. | Weight loss was associated with increased gingival bleeding, showing a peak at 6 months after bariatric surgery. Periodontal pocketing and attachment loss remained unchanged during the first 12 months post surgery. |
| Weinberg et al. | 2018 | Oral health status of patients before and after bariatric bariatric surgery. | To analyze the effect of bariatric surgery on periodontal and dental health and the impact of these changes on quality of life. | Prospective Longitudinal Single-Group Observational | Israel | Medical Clinic(s) / Center(s) / Hospital(s) | 12 months | LAP-Band, Sleeve Gastrectomy, BPD/DS, RYGB | Patients scheduled for all types of bariatric surgeries (gastric band, sleeve gastrectomy, biliopancreatic diversion with duodenal switch or gastric bypass) in the unit of advanced laparoscopic and bariatric surgery within the department of general surgery at Rambam Health Care Campus. | - | Total = 50 Surgery (Before and 6-18 months post op.) (Weight status not disclosed) | 18-60 Mean 38±12 | 52% | X | X | Subjective  Clinical | OHIP-14. DMFT, PlI, calculus, PPD, BOP. | DMFT significantly increased after surgery  The periodontal status did not change between baseline and one year.  Bariatric surgery did not have a significant effect on oral health nor on the perception of quality of life influenced by oral conditions, in spite of significant weight loss and improvements in general health parameters. |
| Karlsson et al. | 2018 | Perceived oral health in patients after bariatric surgery using oral health‐related quality of life measures | To survey the perceived oral health amongst individuals that had undergone bariatric surgery and compare the measures with two cohorts consisting of healthy individuals with respectively at or below versus above a body mass index score of 30. | Cross-sectional | Sweden | (Not mentioned) | - | (Not mentioned) | Overweight - Surgery group: underwent bariatric surgery  Overweight - no Surgery group: BMI ≥30 | BMI <30 | Total = 193 Surgery (Ob/OW) (>1 year post op.) = 77 Non-surgery (Ob/OW) = 45 Non-surgery (NW) = 71 | Surgery (Ob/OW): Mean 43±11   Non-surgery (Ob/OW): Mean 44±11  Non-surgery (NW): Mean 35±10 | 91% | X |  | Subjective | OHIP-S (Swedish version) | Self‐reported oral health problems are more common in individuals that have undergone bariatric surgery than in obese patients and healthy people of normal weight. Individuals that have undergone bar- iatric surgery self‐reported a higher or similar quality of life compared with obese patients; thus, the quality was still lower compared with healthy people of normal weight |
| Aznar et al. | 2019 | Dental wear and tooth loss in morbid obese patients after bariatric surgery | To evaluate the occurrence of dental wear and tooth loss in eutrophic, obese individuals before and after bariatric surgery. | Cross-sectional | Brazil | University Hospital / Academic Departments | - | RYGB | Morbid obese group: BMI ≥ 40 kg/m2  Morbid obese with 24 months of surgery group and with 36 months of  surgery group: underwent bariatric surgery using the gastric by-pass technique, in addition to having undergone only one surgery to treat obesity. | BMI between 18.5-24.99 kg/m2 | Total = 240 Surgery (Ob/OW) (≤ 24 months post op) = 60 Surgery (Ob/OW) (>36 months post op) = 60 Non-surgery (Ob/OW) = 60 Nor-surgery (NW) = 60 | Mean of all groups 36-39 | 80% |  | X | Clinical | IDD, tooth loss. | Dental wear was more prevalent among patients submitted to BGYR, regardless of the postoperative period, and the incisal surfaces of the upper and lower anterior teeth were the most affected. The tooth loss did not show significant differences between the groups. Dental wear was associated with age and number of missing teeth. |
| Balogh et al. | 2020 | Follow-up Study of Microflora Changes in Crevicular Gingival Fluid in Obese Subjects After Bariatric Surgery | To investigate the differences in crevicular gingival fluid microflora between patients in the obese group and the average (normal body weight) population, as well as to assess the effect of weight loss after bariatric surgery on the crevicular microflora. | Prospective Cohort | Hungary | University Hospital / Academic Departments | 6-12 months | RYGB | Patients with obesity visiting the bariatric outpatient office at the University of Debrecen Clinical Department of Internal Medicine or the Department of Surgery for treatment. | Patient with normal body weight who visiting the Faculty of Dentistry at the University of Debrecen | Total = 57 Surgery (Ob/OW) (Before and 6-12 months post op.) = 17 Non-surgery (Ob/OW) = 18 Control (NW) = 22 | 18-58 | 58% |  | X | Clinical  Biologic | PPD, CAL, periodontitis diagnosis. GCF for detection of Actinomyces, Candida, Capnocytophaga, Eikenella, Fusobacterium, Granulicatella, Haemophilus, Lachnoanaerobaculum, Lactobacillus, Micrococcus, Neisseria, Prevotella, Rothia, Staphylococcus, Streptococcus, and Veillonella genera. | After surgery and weight loss, the mean germ count increased, albeit not significantly. Also, Candida albicans and non-albicans Candida species appeared after surgery in subjects where Neisseria was either absent throughout or eliminated after surgery. However, periodontitis did not develop during this time in our subjects. |
| Taghat et al. | 2020 | Oral Health–Related Quality of Life After Gastric Bypass Surgery | To examine how individuals treated for obesity with gastric bypass surgery perceived their oral health and oral health–related quality of life (OHRQoL) | Cross-Sectional | Sweden | Registry | - | RYGB | Underwent GBP surgery in the Region of Västra Götaland, Sweden, during 2011 | - | Total = 644 Surgery (>2 years post op.)  (Weight status not disclosed) | Mean 48±12 | 75% | X |  | Subjective | Self-reported oral health, number of teeth, chewing ability, oral health habits, oral symptoms (including tooth hypersensitivity), acid reflux episodes and vomiting episodes, and OHIP-49 (Swedish version). | A large proportion of individuals having undergone gastric bypass surgery reported problems with their oral health and impacts on their oral health related quality of life |
| Foratori-Junior et al. | 2020 | Evaluation of systemic conditions, tooth loss, body image, and quality of life of women with obesity and women who underwent gastric bypass surgery | To evaluate the systemic conditions, tooth loss, oral health, body image perceptions, and quality of life of women with obesity and women who underwent bypass surgery. | Cross-sectional | Brazil | Medical Clinic(s) / Center(s) / Hospital(s) | - | RYGB | Underwent bariatric surgery within 12 months by the bypass technique, with obesity grades II and III (BMI≥35 kg/m2), with good systemic health and under regular multiprofessional follow-up (for both groups) | - | Total = 60 Surgery (Ob/OW) (≥12 months post op.) = 30 Non-surgery (Ob/OW) = 30 | Surgery (Ob/OW): Mean 47±11  Non-surgery (Ob/OW): Mean 38±11 | 100% | X | X | Subjective  Clinical | Self-reported oral health and oral hygiene behaviors (i.e. frequency of toothbrushing and dental floss use). Tooth loss. | High BMI indicated a higher prevalence of hypertension and a negative impact on oral health perception and quality of life. |
| Vargas et al. | 2020 | Assessment of alveolar bone pattern in obese and non- obese women, before and after bariatric surgery: a prospective cohort study | To elucidate the scientific knowledge about bone pattern and alveolar bone resorption in morbidly obese patients before and after bariatric surgery and to relate them to people with normal weight. | Prospective Cohort | Brazil | University Hospital / Academic Departments | 6 months | (Not mentioned) | Patients with complete permanent dentition up to the left and right lower first molars, those who underwent good quality panoramic and periapical radiographs of the lower premolar region showing a distinct trabecular pattern. Radiographic shots were performed before orthodontic treatment began or two years after orthodontic treatment. | - | Total = 31 Surgery (Ob/OW) (Before and 6 months post op.) = 11 Control (NW) = 20 | 20-35 | 100% |  | X | Radiographic | Radiographic parameters: mandibular cortical index (MCI), mentonian index (MI), panoramic mandibular index (MIP), bone level loss and trabecular pattern evaluation. PI. | The standard alveolar bone presents greater bone loss in obese patients and worsens this standard after bariatric surgery when compared to eutrophic patients. The same happens with the trabecular pattern that becomes sparser after bariatric surgery. |
| Yang et al. | 2021 | Dental Erosion in Obese Patients before and after Bariatric Surgery: A Cross-Sectional Study | To evaluate the presence of dental erosion in obese patients before and after bariatric surgery using the BEWE (basic erosive wear examination) scoring system | Cross-Sectional | Germany | University Hospital / Academic Departments | - | LSG RYGB SADI-S BPD/DS | Underwent bariatric surgery at least 3 months previously. | BMI ≥ 35 kg/m2, and one or more comorbidities (e.g., diabetes, arterial hypertension, or sleep apnea), or BMI ≥ 40 kg/m2 | Total = 62 Surgery (Ob/OW) (≥3 months post op.) = 31 Non-surgery (Ob/OW) = 31 | Surgery (Ob/OW): Mean 43±10  Non-surgery (Ob/OW): Mean 38±10 | 81% |  | X | Clinical | BEWE | No impact on BEWE |
| Alsuhaibani et al. | 2022 | Risk Factors for Dental Erosion After Bariatric Surgery: A Patient Survey | To investigate long-term dietary behaviour and experiences related to symptoms of dental erosion at least 5 years after bariatric surgery. | Cross-sectional | Saudi Arabia | Medical Clinic(s) / Center(s) / Hospital(s) | - | (Not mentioned) | Underwent bariatric surgery at King Saud Medical City 5 to 12 years ago (from 2008 to 2015) | - | Total = 250 Surgery (Ob/OW) (5-12 post op.) | 30- >60 | 69% | X |  | Subjective | Self-reported dental health (i.e. frequency of dental appointments, teeth brushing, use of fluoride toothpaste, use of fluoridated mouth rinse), postoperative oral symptoms (i.e. yellowing of the teeth, chipping, hypersensitivity). | A significant relationship emerged between a high frequency of acidic reflux and a high frequency of oral symptoms related to dental erosion. |
| Tinós et al. | 2022 | impact of bariatric surgery in anxiety and oral conditions of obese individuals: a cohort prospective study | To evaluate the impact of bariatric surgery on anxiety, on initial dental caries lesion and on gingival bleeding in obese patients submitted to bariatric surgery in the public health service. Moreover, the secondary objective was to compare these results with the ones of patients who were not submitted to bariatric surgery. | Prospective Cohort | Brazil | University Hospital / Academic Departments | 12 months | (Not mentioned) | Age between 18 and 60 years, BMI ≥ 40 kg/m2 or 35 kg/m2 with presence of obesity-related comorbidities, history of obesity greater than five years with failure of previous conventional treatments for weight loss, absence of previous bariatric surgery and presence of at least seven dental elements. | - | Total = 89 Surgery (Ob/OW) (Before and 1 year post op.) = 46 Non-surgery (Ob/OW) = 43 | 18-60 | 87% |  | X | Clinical | ICDAS-II, BOP | Significant worsening of the oral condition among the experimental group participants after bariatric surgery, calculations of relative risks and differences between the incidence rates of experimental group and control group showed that the procedure did not represent a risk factor for IDCL nor for gingival bleeding.  The number of teeth with initial dental caries lesion increased significantly after bariatric surgery, as well as the percentage of gingival bleeding and the number of teeth with gingival bleeding |
| Marquezin et al. | 2022 | Impact of gastroplasty on salivary characteristics, dental health status and oral sensory aspects: A controlled clinical study | To evaluate the changes in oral health aspects and saliva composition as a result of the treatment of morbid obesity. | Controlled Clinical | Brazil | Specialized Obesity Treatment / Bariatric Surgery Center | 6 months | Vertical Roux-en-Y Gastroplasty | Patients with morbid obesity (BMI ≥ 40 kg/m2) of both sexes, with at least 20 natural teeth or who use dental prosthesis | - | Total = 73 Surgery (Ob/OW) (Diet program + before and 3- and 6- months post op.) = 39 Non-surgery (Ob/OW) (Before and 3- and 6- months post diet program) = 34 | 19-59 | 82% | X | X | Subjective  Clinical  Biologic | OHIP-14 (Brazilian version), XI (Portuguese version). DMFT, salivary flow/secretion rate, buffering capacity, Total Protein, Alpha-amylase activity. | After 6-months of follow-up, patients undergoing gastroplasty presented an improvement in dietary habits and taste sensitivity. However, increase in the DMFT index was observed over time in both groups. While the salivary flow rate remained relatively stable over time in both groups, a significant decrease in the buffering capacity was observed in the Gastroplasty group. decrease in total protein concentration was observed between 3 and 6 months in the Gastroplasty group and a decrease in alpha-amylase activity was observed after 6 months of follow-up |
| van Leeuwen et al. | 2022 | [Oral health-related quality of life before and after bariatric surgery] | To assess the perceived oral health-related quality of life in dentulous, obese patients and in dentulous, bariatric patients at least one year after they underwent surgery | Cross-sectional | Netherlands | Medical Clinic(s) / Center(s) / Hospital(s) | - | Gastric bypass, sleeve gastrectomy | Patients eligible for bariatric surgery (BMI≥40 kg/m2, BMI≥35 kg/m2 with comorbidity or lower BMI with complication(s) from previous bariatric surgery) | - | Total = 283  Surgery (Ob/OW) (1 year post op.) = 145  Non-surgery (Ob/OW) = 138 | Surgery:  Mean 52±9  Non-surgery (Ob):  Mean 45±12 | 82% | X |  | Subjective | OHIP-14  (Dutch version) | The overall oral health-related quality of life seems unaffected by bariatric surgery: a decrease was however seen regarding the functional limitation subcategory compared to preoperative patients with obesity |
| Čolak et al. | 2022 | Periodontal Therapy in Bariatric Surgery Patients with Periodontitis: Randomized Control Clinical Trial | To determine whether non-surgical periodontal therapy in conjunction with BS improves periodontal and systemic health during the recovery from the surgery. | Randomized Controlled Clinical Trial | Slovenia | University Hospital / Academic Departments | 3-6 months | OAGB RYGB LSG | Severe obesity, ≥18 years old, and with the indication for bariatric surgery treated at the Department of Abdominal Surgery, University Medical Centre, Ljubljana, Slovenia | - | Total = 30 Surgery (Ob/OW) (Before and 3- and 6- months post op.) (With Periodontitis) (Intervention: OHI + Non-Surgical Periodontal Therapy 4 weeks before surgery) = 15 Surgery (Ob/OW) (Before and 3- and 6- months post op.) (With Periodontitis) (Intervention: OHI + Low intensive supra-gingival plaque removal 4 weeks before surgery) = 15 | Mean 51±9 | 70% | X | X | Subjective  Clinical | Self-reported habits (i.e. smoking, alcohol consumption, regular weekly exercise, daily oral hygiene, and regular twice a year dental check-ups), OHIP-14.  PlI, GBI, PPD, BOP, recession, CAL. | Non-surgical periodontal therapy can both prevent further deterioration and improve periodontal health in bariatric surgery patients 3 and 6 months after the surgery. |
| Taghat et al. | 2023 | Impact of Medical and Surgical Obesity Treatment on Dental Caries: A 2-Year Prospective Cohort Study | To compare the effects of bariatric surgery with medical treatment of obesity on dental caries estimates from before until 2 years after treatment. | Prospective Cohort | Sweden | Specialized Obesity Treatment / Bariatric Surgery Center | 2 years | RYGB LSG | Age >18 years  BMI ≥40 kg/m2 or, if obesity-related comorbidities were present, BMI ≥35 kg/m2, Sufficient knowledge of the Swedish language. | - | Total = 66 Surgery (Ob/OW) (Before and 2 years post op.) = 40 Non-surgery (Ob/OW) (Before and 2 years post diet program) = 26 | Surgery (Ob/OW): Mean 29±5  Non-surgery (Ob/OW): Mean 28±5 | 100% | X | X | Subjective  Clinical | Self-reported toothbrushing, interdental cleaning, visits to the dentist the last 5 years, and reason for the most recent dental appointment. ICDAS-II, plaque, BOP. | Two years after obesity treatment, a significant increase in dental caries was registered in the surgically treated but not in the medically treated women. |
| Ribeiro et al. | 2023 | Bypass gastroplasty impacts oral health, salivary inflammatory biomarkers, and microbiota: a controlled study | To evaluate the oral health status, salivary inflammatory mark- ers, and microbiota in patients undergoing gastroplasty, comparing with a sex-matched control group undergoing dietary advice. | Prospective Cohort | Brazil | Medical Clinic(s) / Center(s) / Hospital(s) | 6 months | Vertical Roux-en-Y Gastroplasty | Obesity grades II or III (BMI ≥ 35 kg/m2), both sexes, with at least 20 natural teeth | - | Total = 40 Surgery (Ob/OW) (Diet program + before and 3- and 6- months post op.) = 20 Non-surgery (Ob/OW) (Before and 3- and 6- months post diet program) = 20 | Surgery (Ob/OW): Mean 32±6  Non-surgery (Ob/OW): Mean 35±7 | 75% |  | X | Clinical  Biologic | DMFT, CPI, salivary flow/secretion rate, buffering capacity, salivary cytokines concentrations of IL-6, IL-10, TNF-α, and IFNy and salivary microbiota. | Caries activity increased in both groups, and the gastroplasty group showed worse periodontal status after three months. IFNγ and IL10 levels decreased in the gastroplasty group at 3 months, while a decrease was observed in the control group at 6 months; IL6 decreased in both groups. Salivary flow, buffering capacity and TNFα did not change. |
| Marsk et al. | 2024 | Metabolic surgery and oral health: A register-based study | To investigate the impact of metabolic surgery on the risk for dental interventions. | Retrospective Cohort | Sweden | Registry | 10 years | RYGB LSG | All individuals in SOReg having metabolic surgery with either gastric bypass or sleeve gastrectomy between January 1, 2009 and December 31, 2018 | A reference cohort from the general population matched for age, gender, and county of residence at the time of case patient exposure, that is, metabolic surgery (matching ratio 1:10) | Total = 590,073 Surgery (Ob/OW) (0-10 years post op.) = 53,643 Control (NW) = 536,430 | Mean 41±11 | 76% |  | X | Clinical | Incidence of tooth extractions, restorative interventions, endodontic interventions and periodontal interventions. | Metabolic surgery seems to have a substantial negative impact on oral health. |
| Kogawa et al. | 2024 | The changes on salivary flow rates, buffering capacity  and chromogranin A levels in adults after bariatric surgery | To investigate changes in salivary flow rates, buffering capacity, and salivary chromogranin A (CHGA) levels in adults undergoing bariatric surgery (BS) compared with a non-obese control group. | Cross-sectional | Brazil | Medical Clinic(s) / Center(s) / Hospital(s) | - | RYGB LSG | Individuals aged over 50 who had undergone bariatric surgery at least one year prior to the study. | - | Total = 62  Surgery (Ob/OW) (≥1 year post op.) = 31  Control (NW) = 31 | Median 60 | 87% | X | X | Subjective  Biologic | Self-reported xerostomia,  salivary flow/secretion, buffering capacity and chromogranin A. | The bariatric surgery group exhibited higher prevalence of self-reported xerostomia and tongue dryness compared to control.  No significant differences were observed between groups regarding salivary secretion rates (unstimulated, stimulated or upper labial saliva) and the prevalence of hyposalivation.  The buffering capacity in the bariatric surgery group was significantly lower than that in the control group. |

^a^ Country of study execution

^b^ Settings in which study was performed

^c^ BPD/DS - Biliopancreatic Diversion With Duodenal Switch; DS - Duodenal Switch; JI Bypass - Jejunoileal Bypass; LAP-Band - Laparoscopic Adjustable Gastric Banding OR Gastric Band Surgery; LSG - Laparoscopic Sleeve Gastrectomy OR Vertical Sleeve Gastrectomy OR Gastric Sleeve; OAGB - One Anastomosis Gastric Bypass; RYGB - Roux-en-Y Gastric Bypass OR Laparoscopic Roux-en-Y Gastric Bypass; SADI-S - Single Anastomosis Duodeno-Ileal Bypass with Sleeve Gastrectomy; SRVG - Silastic Ring Vertical Gastroplasty;

^d^ Ob - Obese; OW - Overweight; NW - Normal Weight; Post Op. - Post Operation

^e^ BEWE - Basic Erosive Wear Examination (Bartlett et al., 2008); BOP - Bleeding on Probing; CAL - Clinical Attachment Level/Loss; CI - Calculus Index (Ainamo & Bay, 1975); CPI - Community Periodontal Index; DMFT - Number of Decayed, Missing and Filled Teeth; DWI - Dental Wear Index (S. H. dC. Sales-Peres et al., 2008); GBI - Gingival Bleeding Index; GCF - Gingival Crevicular Fluid; GI - Gingival Index (Löe & Silness, 1963); HRQoL - Health-Related Quality of Life; ICDAS - International Caries Detection and Assessment System (Ismail et al., 2008); IDD - Tooth Wear Index (S. H. dC. Sales-Peres et al., 2013); OIDP - Oral Impact affecting Daily Performance (Adulyanon et al., 1996); OHIP - Oral Health Impact Profile (Slade & Spencer, 1994); PI - Plaque Index (Turesky et al., 1970); PlI - Plaque Index (Silness & Löe, 1964); PPD - Probing Pocket Depth; SBI - Sulcus Bleeding Index (Mühlemann & Son, 1971); XI - Xerostomia Inventory (Thomson et al., 1999);
