# Supplementary material for: Oral Health in Individuals After Bariatric Surgery: A Systematic Scoping Review
Source: Obes Surg. 2025 Mar 19;35(5):1878–99. doi: 10.1007/s11695-025-07793-w (PMC12065770; doi:10.1007/s11695-025-07793-w)
Supplement: Supplementary file 5 — Supplementary file5 (DOCX 144 KB) [file 11695_2025_7793_MOESM5_ESM.docx]

Appendix 5: Systematic reviews included in the scoping review (n=9).

| Authors | Year | Study Title | Study Objective(s) | Study Type | Country^a^ | Settings^b^ | Study Duration | Type of Bariatric Surgery^c^ | Inclusion criteria | Number of Participants^d^ | Age (years) | Sex  (F%) | Studied Variables Category | | Variables Sub-Category | Studied Outcome Variable(s)^e^ | Key findings related to scoping review question |
| --- | --- | --- | --- | --- | --- | --- | --- | --- | --- | --- | --- | --- | --- | --- | --- | --- | --- |
|  |  |  |  |  |  |  |  |  |  |  |  |  | Subjective | Objective |  |  |  |
| Salgado-Peralvo et al. | 2018 | Bariatric surgery as a risk factor in the development of dental caries: a systematic review | To know whether those obese patients who have undergone bariatric surgery have a greater risk of developing dental caries. | Systematic review  (Without meta-analysis) | Spain | (Not mentioned) | 6-24 months | RYGB, LSG | Studies done with humans, articles published in English and Spanish, series of cases, clinical trials. | 539 participants 9 articles | 36-45 | 81% |  | X | Clinical  Biologic | DMF, salivary microbiology, salivary flow | Patients who have undergone bariatric surgery have a greater risk of dental caries. |
| de Souza et al. | 2018 | Relationship between bariatric surgery and periodontal status: a systematic review and meta-analysis | To investigate the effect of bariatric surgery on periodontal status. | Systematic review with meta‐analysis | Brazil | (Not mentioned) | 1-12 months | RYGB | Observational studies (case-control, cross-sectional, and cohort) that reported periodontal outcomes before and after bariatric surgery among patients aged ≥18 years who had undergone any type of bariatric surgery. | 902 participants  qualitative analysis:  9 articles  meta-analysi:  5 articles | 35-47 | 82% |  | X | Clinical | PPD, BOP PI, CAL, GI | Bariatric surgery may improve the periodontal status of patients with obesity, mainly plaque index. |
| Fontanille et al. | 2018 | Bariatric surgery and periodontal status: A systematic review with meta-analysis | To evaluate the current literature on the impact of bariatric surgery on periodontal status. | Systematic review with meta‐analysis | France | (Not mentioned) | 6-12 months | RYGB | Cross-sectional and prospective studies dealing with bariatric surgery that met the following criteria: adult patients with obesity aged ≥18 years at baseline; follow-up after bariatric surgery ≥6 months, available periodontal records | 1159 participants  qualitative analysis:  10 articles  meta-analysi:  4 articles | 35±9 - 49±9 | 76% |  | X | Clinical | BOP, PI, PPD, CAL, suppuration, missing teeth. | Deterioration of periodontal status may be observed in the first 6 months after surgery. |
| dos Santos et al. | 2019 | Clinical periodontal conditions in individuals after bariatric surgery: a systematic review and meta-analysis | To assess whether bariatric surgery has any influence on the clinical periodontal conditions in obese patients. | Systematic review with meta‐analysis | Brazil | (Not mentioned) | 3-12 months | RYGB, LAP-Band, LSG, BPD/DS | Randomized controlled trials, prospective and retrospective studies, observational studies, longitudinal studies, and cohort studies dealing with bariatric surgery with at least 3 months of follow-up. Only adult patients with obesity who were 18 years of age at baseline, had undergone bariatric surgery, had available periodontal records | 547 participants  qualitative analysis:  6 articles  meta-analysi:  4 articles | 18-60 | 62% |  | X | Clinical | BOP, PPD, CAL, CPI | Bariatric surgery does not influence BOP or PPD, but leads to a worsening of CAL. |
| Farias et al. | 2019 | Influence of Bariatric Surgery on Salivary Flow: a Systematic Review and Meta-Analysis | To investigate the relationship between bariatric surgery and the possible alterations in salivary flow in morbidly obese individuals undergoing this type of treatment. | Systematic review with meta‐analysis | Brazil | (Not mentioned) | 6-24 months | RYGB, LSG, laparoscopy | Population: individuals with morbid obesity.  Intervention: in individuals with morbid obesity undergoing bariatric surgery  Comparison: morbidly obese subjects not submitted to bariatric surgery. Outcome: oral health condition (saliva).  Randomized clinical trials, prospective, retrospective, longitudinal, cohort, transverse studies. | 409 participants  qualitative analysis:  0 articles  meta-analysi:  8 articles | 26–55 | 83% |  | X | Biologic | Salivary flow | There is no significant change in salivary flow in patients who underwent bariatric surgery and were followed up for up to 24 months. |
| Castilho et al. | 2019 | Bariatric surgery impact on gastroesophageal reflux and dental wear: a systematic review | To evaluate the occurrence of tooth wear after bariatric surgery by means of a systematic review of the literature. | Systematic review  (Without meta-analysis) | Brazil | Medical Clinic(s) / Center(s) / Hospital(s) | 3-6 months | 3 studies: RYGB  Others:  Not mentioned | Cross-sectional, longitudinal clinical studies. | 379 participants  4 articles | 41±10 | (Not mentioned - majority were women) | X | X | Subjective  Clinical | DWI, Self-reported dental hypersensitivity | Association between tooth wear, gastroesophageal reflux and bariatric surgery. |
| Quintella et al. | 2020 | Relationship between bariatric surgery and dental erosion: a systematic review | To evaluate whether bariatric surgery presented a greater risk of dental erosion. | Systematic review  (Without meta-analysis) | Brazil | (Not mentioned) | 3 months-4 years | RYGB | Randomized controlled trials, prospective and retrospective studies, observational studies, cross-sectional studies, longitudinal studies, and cohort studies dealing with bariatric surgery. Only adult patients with obesity 18 years or older at baseline and those who underwent bariatric surgery with available dental records. | 441 participants 5 articles | 20-70 | 52% |  | X | Clinical  Biologic | DWI, DMFT, salivary flow, tooth sensibility | Patients undergoing bariatric surgery had a higher incidence of dental erosion. |
| Čolak et al. | 2021 | The effect of bariatric surgery on periodontal health: systematic review and meta-analyses | To evaluate all existing research outputs on the periodontal consequences of bariatric surgery procedures. | Systematic review with meta‐analysis | Slovenia | (Not mentioned) | 6-12 months | (Not mentioned) | studies conducted on humans, published in the English language, including early view articles from journals, with the date of the publication between 2000 and 2019, with access to the full-text article.  Population: obese patients undergoing BS, older than 18 years, both sexes, who voluntarily gave consent to be a part of the study, and who received clinical periodontal examinations before the surgery and at the follow-up. Prospective cohort studies on the influence of BS on the periodontal status, with data recorded before and at least once within 6 to 12 months after BS. | qualitative analysis:  4 articles  meta-analysi:  4 articles | (Not mentioned) | (Not mentioned) |  | X | Clinical | BOP, CAL, PPD | Bariatric surgery may lead to a short-term worsening of periodontal status 6 months after bariatric surgery, which is not present 12 months after bariatric surgery. |
| Ferraz et al. | 2023 | Impact of bariatric surgery on oral health: a systematic review and meta‐analysis | To evaluate the impact of bariatric surgery on the oral health status of obese individuals. | Systematic review with meta‐analysis | Brazil | (Not mentioned) | 1-48 months | LAP-Band, RYGB, LSG, gastric bypass with omega loop, laparoscopic gastric plication, LSG | Adult individuals (> 18 – 60 years of age) with an indication for bariatric surgery. The studies should have compared pre- and postoperative moments (paired or unpaired) and should have evaluated at least one of the following aspects: dental aspects, periodontal aspects, masticatory function, flow, and salivary composition.  Randomized, non-randomized, quasi-experimental or pseudo-randomized clinical trials, observational cohort, case–control or cross-sectional studies. | 2227 participants 30 articles | 16-70 | (Not mentioned) |  | X | Clinical  Biologic | PPD, GBI, wear, caries, hypersensitivity, chewing, alveolar bone loss, halitosis, salivary flow | Patients undergoing bariatric surgery may show no difference when considering the rate of caries and tooth loss and little or no effect on salivary flow and PPD compared to obese patients that did not undergo bariatric surgery. On the other hand, greater attention should be given to dentin wear in post-surgical patients. |

^a^ Country of research team or principal investigator.

^b^ Settings in which included studies were carried out.

^c^ BPD/DS - Biliopancreatic Diversion With Duodenal Switch; LAP-Band - Laparoscopic Adjustable Gastric Banding OR Gastric Band Surgery; LSG - Laparoscopic Sleeve Gastrectomy OR Vertical Sleeve Gastrectomy OR Gastric Sleeve; RYGB - Roux-en-Y Gastric Bypass OR Laparoscopic Roux-en-Y Gastric Bypass.

^d^ Total number of participants from included studies.

^e^ BOP - Bleeding on Probing; CAL - Clinical Attachment Level/Loss; CPI - Community Periodontal Index; DMFT - Number of Decayed, Missing and Filled Teeth; DWI - Dental Wear Index (S. H. dC. Sales-Peres et al., 2008); GBI - Gingival Bleeding Index; ICDAS - International Caries Detection and Assessment System (Ismail et al., 2008); PI - Plaque Index (Turesky et al., 1970); PlI - Plaque Index (Silness & Löe, 1964); PPD - Probing Pocket Depth.
